# Therapie des Xanthoma disseminatum – eine systematische Literaturrecherche

**DOI:** 10.1111/ddg.15824_g

**Published:** 2025-09-15

**Authors:** Inga Hansen‐Abeck, Mina Hillemans, Finn Abeck, Stefan W. Schneider, Nina Booken

**Affiliations:** ^1^ Klinik und Poliklinik für Dermatologie und Venerologie Universitätsklinikum Hamburg‐Eppendorf Hamburg Deutschland

**Keywords:** non‐Langerhans cell histiocytosis, therapy, Xanthoma disseminatum, Nicht‐Langerhans‐Zell‐Histiozytose, Therapie, Xanthoma disseminatum

## Abstract

Das Xanthoma disseminatum ist eine seltene Erkrankung aus dem Spektrum der Nicht‐Langerhans‐Zell‐Histiozytosen, welche in drei Gruppen eingeteilt werden kann und teilweise mit einer Systembeteiligung einhergeht. Da die Erkrankung selten ist, fehlen standardisierte Therapierichtlinien, wodurch die Behandlung im klinischen Alltag erschwert wird. Ziel dieser Arbeit war es daher, mittels systematischer Literaturrecherche die Therapieerfahrungen der letzten 22 Jahre zusammenzufassen und einen Überblick über die verschiedenen Behandlungsoptionen zu geben.

Es wurde eine systematische Literaturrecherche zum Thema Xanthoma disseminatum und Therapiestrategien in der Datenbank PubMed/MEDLINE für den Zeitraum vom 26.06.2002 bis 26.06.2024 durchgeführt. Diese Recherche ergab 38 Treffer, von denen 19 in die Synthese eingeschlossen wurden. Die beschriebenen Therapien umfassen immunsuppressive, zytostatikabasierte, lipidsenkende, operative sowie UV‐strahlen‐ und laserbasierte Ansätze. Am häufigsten wurde über die Therapie mit Cladribin berichtet.

Die aktuelle Datenlage basiert überwiegend auf Fallberichten und Fallserien, welche in dieser Übersicht dargestellt und zusammengefasst wurden. Sie kann somit als Grundlage für Therapieentscheidungen herangezogen werden.

## EINLEITUNG

Das Xanthoma disseminatum (XD) ist eine seltene Erkrankung, die 1938 erstmals von Montgomery und Osterberg beschrieben wurde. Sie gehört zu den benignen Nicht‐Langerhans‐Zell‐Histiozytosen.^1^, ^2^, ^3^ Das Alter bei Erkrankungsbeginn liegt zwischen 8 Monaten und 85 Jahren, wobei die Erkrankung meist im jungen Erwachsenenalter auftritt und Männer häufiger betroffen sind als Frauen.^2^, ^4^, ^5^ Gekennzeichnet ist die Erkrankung durch die ätiologisch bislang ungeklärte Proliferation histiozytärer Zellen sowie sekundäre Lipidablagerungen in der Dermis und in inneren Organen.^1^, ^2^, ^6^

**ABBILDUNG 1:**
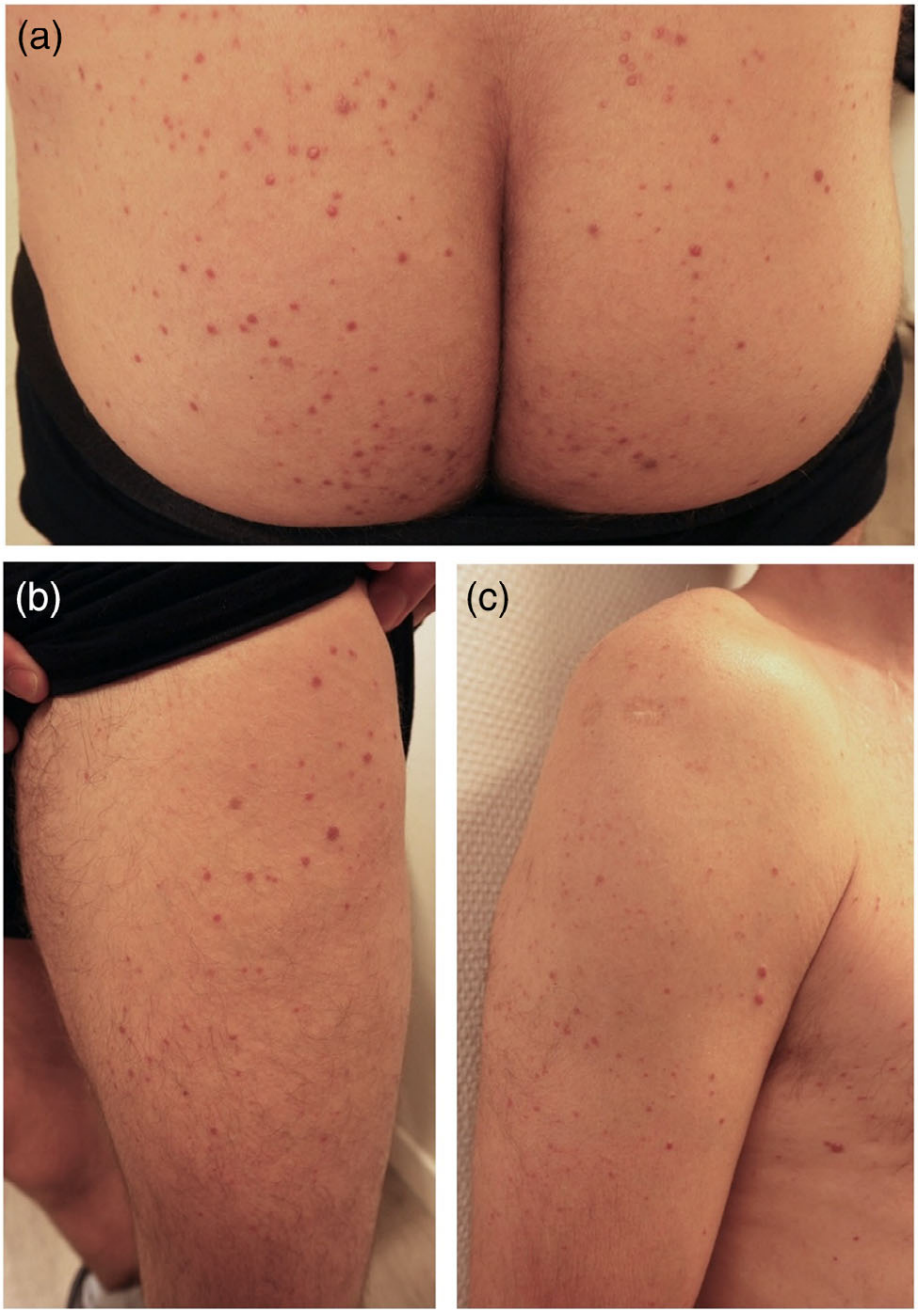
Klinisches Bild eines Xanthoma disseminatum mit disseminierten, diffus verteilten rötlich‐bräunlichen Papeln (a) gluteal (b) am Bein und (c) am Arm.

Klinisch werden drei Manifestationsformen des XD unterschieden: eine selbstlimitierende Form, eine persistente, rein kutane Form und die progressive systemische Form mit Organdysfunktion und möglicher Beteiligung des zentralen Nervensystems (ZNS).^4^ Die kutanen Manifestationen äußern sich durch schleichendes Auftreten disseminierter, kutaner, gelblich‐roter oder rötlich‐bräunlicher Papeln (Abbildung 1).^7^ Charakteristische Lokalisationen sind die Augenlider sowie die Intertrigines und Beugeseiten der Extremitäten.^2^, ^8^ Im Verlauf kommt es oftmals zur Ausbreitung der Läsionen, welche teilweise zu Plaques konfluieren.^9^ Etwa 30–40 % der Fälle gehen mit Schleimhautbeteiligung einher, häufig betroffen sind dabei Oropharynx, Kehlkopf und Bindehaut, wodurch es zu Dysphagie, Obstruktion der Atemwege sowie Erblindung kommen kann.^7^ Die progressive systemische Form wird in 40 % der Fälle von Diabetes insipidus (DI) begleitet, welcher durch Veränderungen in der Hypothalamus‐Hypophysen‐Region verursacht wird und oftmals irreversibel ist.^7^

**ABBILDUNG 2:**
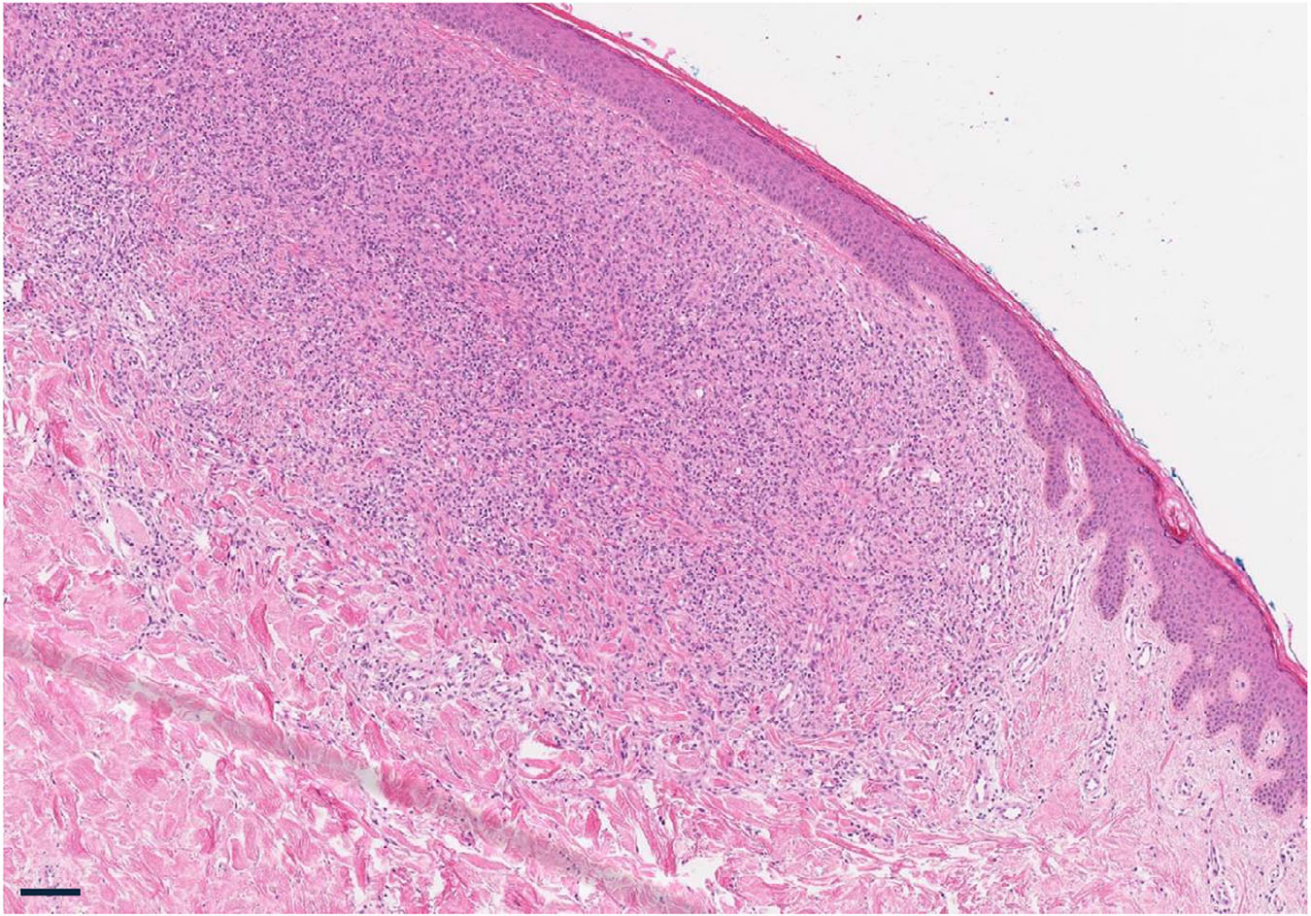
Histologisches Bild: Epidermis mit abgeflachten Reteleisten, dichte Infiltrate aus Makrophagen und Lymphozyten mit wenigen Eosinophilen (Hämatoxylin‐Eosin‐Färbung, Maßstab: 250 µm).

Histologische und immunhistochemische Untersuchungen sind für die Diagnosestellung des XD notwendig.^10^ Histopathologisch besteht im Anfangsstadium eine Infiltration von Makrophagen mit dendritischer Form, es finden sich nur wenige Schaumzellen, Lymphozyten und eosinophile Granulozyten (Abbildung 2).^7^ Im späteren Stadium der Erkrankung überwiegen in der Regel xanthomatisierte Makrophagen vom Touton‐Typ mit mehrkernigen Riesenzellen, welche die Dermis flächig infiltrieren.^7^ Immunhistochemisch sind die Oberflächenmarker CD68 und Faktor XIIIa der Makrophagen bedeutsam.^6^, ^7^ Die Marker S‐100, CD1a und Birbeck‐Granula sind in Abgrenzung zu den Langerhans‐Zell‐Histiozytosen in der Regel negativ.^6^, ^7^ Zum Ausschluss möglicher Systembeteiligungen können auch computertomographische Untersuchungen des Thorax, magnetresonanztomographische Untersuchungen des Kopfes, laryngoskopische und ophthalmologische Untersuchungen durchgeführt werden.^8^, ^11^, ^12^

Da Therapiestandards für das XD fehlen, war es Ziel dieser Arbeit, die publizierten Therapieansätze systematisch zusammenzufassen.

## METHODIK

Es erfolgte eine systematische Literaturrecherche mit den Suchbegriffen „(xanthoma disseminatum) [title/abstract] AND (therapy) [all fields]“. Die Literaturrecherche wurde in der Datenbank PubMed/MEDLINE für den Zeitraum vom 26.06.2002 bis 26.06.2024 durchgeführt. Die Treffer wurden im Anschluss in einer Ergebnistabelle in Microsoft Excel dokumentiert. In einem ersten Screeningdurchgang wurden die Ergebnisse anhand ihres Titels und Abstracts untersucht. Unpassende Ergebnisse wurden anhand im Vorfeld festgelegter Ein‐ und Ausschlusskriterien aussortiert (Tabelle 1). Die verbliebenen Treffer wurden im zweiten Durchgang anhand des Volltextes durchsucht. Im Anschluss wurden die relevanten Informationen aus den eingeschlossenen Publikationen extrahiert und in einer zweiten Ergebnistabelle dokumentiert. Das Screening wurde dabei von zwei Personen (M.Z. und I.H.‐A.) unabhängig durchgeführt. Abweichungen wurden im Nachgang diskutiert. Zudem wurden die Literaturverzeichnisse der eingeschlossenen Arbeiten hinsichtlich weiterer relevanter Studien durchsucht. Die Arbeit wurde gemäß den Prinzipien der *Preferred Reporting Items for Systematic Reviews and Meta‐Analyses* (PRISMA) durchgeführt.

**TABELLE 1:** Ein‐ und Ausschlusskriterien.

Einschlusskriterien	Ausschlusskriterien
***Screening Titel und Abstract***	
Arbeit untersucht den Zusammenhang zwischen XD und einer Therapie	Unpassende Thematik Sonderformen eines XD
Publikationszeitraum: 26.05.2002 bis 26.05.2022	Unpassende Methodik
Sprache: Deutsch oder Englisch	Erkrankungsbeginn vor dem 18. Lebensjahr
***Volltextscreening***	
	Keine kutanen Manifestationen oder Therapie beschrieben Kein Volltext verfügbar oder anforderbar
	Sprache nicht Deutsch oder Englisch

## ERGEBNISSE

**ABBILDUNG 3:**
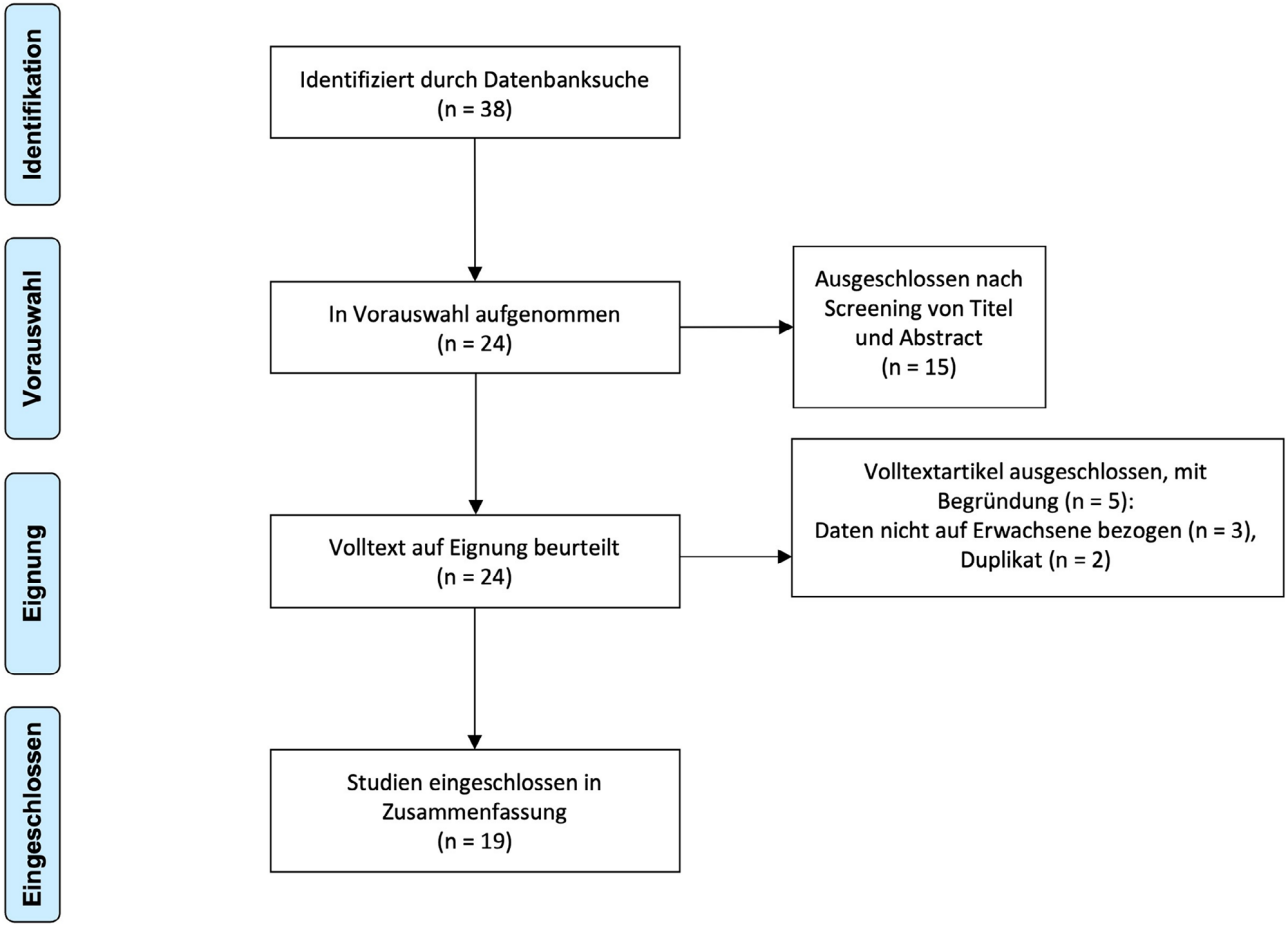
PRISMA Flow‐Chart.

Die systematische Literaturrecherche ergab 39 Arbeiten in PubMed/MEDLINE. Insgesamt 15 Publikationen konnten im ersten Screeningdurchgang ausgeschlossen werden (Abbildung 3). Die verbliebenen 24 Publikationen wurden im zweiten Durchgang anhand ihres Volltextes auf ihre Eignung überprüft. Vier weitere Arbeiten wurden hierbei ausgeschlossen, sodass insgesamt 20 Arbeiten in die Analyse eingeschlossen wurden, welche über 25 Patienten mit XD berichteten. Eine Übersicht der Ergebnisse aus den eingeschlossenen Publikationen ist in Tabelle 2 dargestellt.

**TABELLE 2:** Ergebnistabelle der eingeschlossenen Arbeiten zum Xanthoma disseminatum.

Erstautor, Publikationsjahr, *Methodik*	Klinisches Bild und Patientencharakteristika	Therapieansatz	Ergebnis
Gayed et al., 2024 *Fallbericht*	**Persistentes XD, m, 65 Jahre** *Kutane Manifestation*: ‐ Effloreszenz: rot‐bräunliche Papeln, teilweise konfluierend zu Plaques ‐ Lokalisation: Gesicht, Stamm, obere Extremität, Axillae	*Cladribin*: 6 Zyklen, 0,14 mg/kg/Tag für 5 Tage pro Monat	Nahezu komplette Remission der kutanen Manifestationen
Jung et al., 2024 *Fallbericht*	**Progressive systemisches XD, m** *Kutane Manifestation*: ‐ Effloreszenz: bräunliche Papeln und Plaques ‐ Lokalisation: Gesicht unter Aussparung der Nase, vereinzelt intertriginös *Systembeteiligung*: DI	*Pulsed‐Dye‐Laser*: ‐ Initial: 0,45 ms, 7 mm, 7 J/cm2, 1 pass, 1 x monatlich ‐ Im Verlauf: bis zu 0,45 ms, 10 mm, 10 J/cm2, 1 pass, bis zu 5 x monatlich *Vasopressin (DI)*	Gute partielle Remission: kutane Manifestationen nach 6 Monaten Behandlung
Zhou et al., 2023 *Fallserie*	**Progressive systemisches XD, m, 20 Jahre** *Kutane Manifestation*: ‐ Effloreszenz: bräunliche Papeln und Plaques ‐ Lokalisation: Gesicht inkl. Augenlider, Intertrigines, Stamm, Genitalregion, Oberschenkel, Kniekehlen, Mundschleimhaut *Systembeteiligung*: Dyspnoe	*Cladribin*: 6 Zyklen, 0,14 mg/kg/Tag für 5 Tage pro Monat	*Mukokutane Manifestation: p*artielle Remission (> 70 %) *Systembeteiligung: p*artielle Remission
	**Progressive systemisches XD, w, 47 Jahre** *Kutane Manifestation*: ‐ Effloreszenz: bräunliche Papeln und Plaques ‐ Lokalisation: Gesicht inkl. Augenlider, Intertrigines, Oberschenkel, Ellenbeugen, Kniekehlen, Mundschleimhaut *Systembeteiligung*: Dyspnoe	*Cladribin*: 5 Zyklen, 0,14 mg/kg/Tag für 5 Tage pro Monat	*Mukokutane Manifestation: p*artielle Remission (> 80 %) *Systembeteiligung*: Komplettremission
García‐Legaz et al., 2021 *Fallbericht*	**Persistentes XD, w, 40 Jahre** *Kutane Manifestation*: ‐ Effloreszenz: multiple, schnell progressive, gelb‐bräunliche Papeln ‐ Lokalisation: Gesicht, obere Extremitäten ‐ Symptomatik: Juckreiz	*Schmalspektrum‐UV‐B‐ Phototherapie*: (3 Tage/Woche): 54 Bestrahlungen, kumulative Dosis: 62,14 J/cm^2^	Partielle Remission, rasche Juckreizlinderung, keine Nebenwirkungen
Tuan et al., 2019 *Fallserie*	**Progressiv systemisches XD, m, 34 Jahre** *Kutane Manifestation*: ‐ Effloreszenz: gelbliche Papeln und Nodi ‐ Lokalisation: Gesicht inkl. Augenlider, Intertrigines, Genitalregion, Perianalregion, Mundschleimhaut *Systembeteiligung*: DI	*Cladribin*: 5–9 Zyklen, 0,14 mg/kg/Tag für 5 Tage pro Monat	*Mukokutane Manifestation*: Remission *Systembeteiligung*: keine Aussage
	**Progressiv systemisches XD, m 35 Jahre** *Kutane Manifestation*: ‐ Effloreszenz: gelbliche Papeln und Nodi ‐ Lokalisation: Gesicht inklusive Augenlider, Intertrigines, Genitalregion, Perianalregion, Mundschleimhaut *Systembeteiligung*: DI	*Mukokutane Manifestation*: Remission *Systembeteiligung: k*eine Aussage
Al‐Tarcheh et al., 2019 *Fallbericht*	**Progressiv systemisches XD, w, 24 Jahre** *Kutane Manifestation*: ‐ Effloreszenz: gelb‐braune Papeln, Durchmesser: 1–7 mm ‐ Lokalisation: Gesicht inklusive Augenlider, Stamm, Axillae *Systembeteiligung*: Gelenkschmerzen, DI, Lunge (zentrilobuläres Emphysem)	*Cladribin*: 6 Zyklen, 0,14 mg/kg/Tag für 5 Tage pro Monat *Desmopressin (DI)*	*Remission*: Respirationstrakt, Gelenkschmerzen *Kutane Manifestation*: Stabilisierung
Sawatkar et al., 2016 *Fallbericht*	**Progressiv systemisches XD, m, N/A** *Kutane Manifestation*: ‐ Effloreszenz: Multiple, hautfarbene bis gelbliche Papeln und Nodi, teilweise zu Plaques konfluierend ‐ Lokalisation: Gesicht, Intertrigines, Mundschleimhaut *Systembeteiligung*: DI	*Imatinib*: ‐ initial 100 mg/Tag (oral) ‐ progressive Steigerung auf 400 mg/Tag	*Remission: m*ukokutane Manifestationen, DI
Gupta et al., 2015 *Fallbericht*	**Progressiv systemisches XD, m, 23 Jahre** *Kutane Manifestation*: ‐ Effloreszenz: gelblich‐bräunliche Papeln, teilweise zu Plaques konfluiert, braune Maculae ‐ Lokalisation: Gesicht, Kinn, Nacken, Axillae *Systembeteiligung*: DI	*Cladribin*: 8 Zyklen, 0,14 mg/kg/Tag für 5 Tage pro Monat *Desmopressin (DI)*	*Remission: a*lle kutanen Manifestationen
Campero et al., 2016 *Fallbericht*	**Progressiv systemisches XD, m, 32 Jahre** *Kutane Manifestation*: ‐ Effloreszenz: disseminierte, gelb‐bräunliche Papeln ‐ Lokalisation: Augenlider, Stamm, Axillae *Systembeteiligung*: ‐ ZNS: Panhypopituitarismus mit DI und Oligozoospermie sowie Temporallappenepilepsie	*Strahlentherapie*: Temporallappens und Hypophyse: 38 Gy Gesamtdosis*Anakinra*: ‐ Initial 100 mg s.c./Tag, über 15 Monate ‐ Dann 100 mg s.c./Woche über 24 Monate ‐ Dann 100 mg s.c./Monat seit 12 Monaten) *Antikonvulsive Therapie* (Epilepsie)	*Strahlentherapie: sine effectu* *Anakinra*: Remission der kutanen Manifestation und der ZNS‐Beteiligung (unter fortlaufender antikonvulsiver Therapie)
Hsu et al., 2014 *Fallbericht*	**Persistentes XD, m, 70 Jahre** *Kutane Manifestation*: ‐ Effloreszenz: Knötchen ‐ Lokalisation: Gesicht, Mundschleimhaut, Intertrigines, Genitalien, mediale Oberschenkel	*Exzision* *Non‐ablativer 1450 nm‐Diodenlaser*: 3 Laserbehandlungen im Abstand von 14 Tagen über 6 Wochen im Gesicht, Einzeldosis: 18 J/cm^2^ x 25 ms	*Exzision*: Rezidiv *Non‐ablativer 1450‐nm Diodenlaser*: Remission der kutanen Manifestationen im Gesicht
Park et al., 2014 Fallbericht	**Selbstlimitierendes XD, m, 61 Jahre** *Kutane Manifestation*: ‐ Effloreszenz: multiple, runde bis ovale, rosa‐bräunliche Papeln, Durchmesser: bis 0,5 cm ‐ Lokalisation: Stamm, obere Extremität	*Doxycyclin*: 220 mg/Tag für 1 Monat *Ciclosporin*: 300 mg/Tag für 6 Wochen	*Doxycyclin und Ciclosporin: k*eine Veränderung Im Verlauf selbstlimitierend
Mahajan et al., 2013 *Fallbericht*	**Persistentes XD, m, 18 Jahre** *Kutane Manifestation*: ‐ Effloreszenz: multiple, gruppiert stehende, orange‐braune, papulonoduläre Läsionen mit glatter Oberfläche ‐ Lokalisation: Gesicht inklusive Augenlieder, Hals, Axillae, Ellenbeugen, Kniekehlen	*Azathioprin*: 50 mg, 2 x täglich In Kombination mit *Prednisolon*: 40 mg alle 2 Tage	*Kutane Manifestationen*: Stabilisierung, teilweise Remission
Lee et al. 2011 *Fallbericht*	**Progressiv systemisches XD, w, 63 Jahre** *Kutane Manifestation*: ‐ Effloreszenz: gelb‐bräunliche erbsengroße Papeln; stellenweise konfluierende, gelbliche Plaques ‐ Lokalisation: Gesicht inklusive Augenlieder, Zunge, Brust, Nacken, Intertrigines, Genitalbereich, Ellenbeugen, Kniekehlen *Systembeteiligung*: Miktionsverhalt, Dyspnoe und Dysphagie	*Cyclophosphamid*: ‐ 50–100 mg täglich für 2 Jahre *Kombinationstherapie (4 Monate)*: ‐ Rosiglitazon (4 mg/Tag) ‐ Simvastatin (10 mg/Tag) ‐ Acipimox (500 mg/Tag) *Im Verlauf Simvastatin‐Monotherapie*:10 mg/Tag für 5 Monate	*Cyclophosphamid: f*ehlendes Ansprechen *Lipidsenker*: Remission aller Hautläsionen, Dyspnoe, Dysphagie und Miktionsverhalt
Khezri et al., 2010 *Fallserie mit 8 Patienten, von denen 5 eine Behandlung erhielten und in dieses Review eingeschlossen wurden*.	**(1) Persistentes XD, m 55 Jahre** *Kutane Manifestation*: ‐ Effloreszenz: gelb‐orange Papeln und Plaques ‐ Lokalisation: Augenlider, Stamm, proximale Extremitäten	*Cladribin*: 8 Zyklen 0,14 mg/kg/KG i.v., über 5 Tage, je ein Zyklus/Monat	Komplette Remission
	**(2) Persistentes XD, m, 46 Jahre** *Kutane Manifestation*: ‐ Effloreszenz: gelbliche Papeln ‐ Lokalisation: Gesicht inklusive Augenlider, Stamm	*Cladribin*: 5 Zyklen 0,14 mg/kg/Tag für 5 Tage pro Monat	Nahezu komplette Remission
	**(3) Persistentes XD, m, 41 Jahre** *Kutane Manifestation*: ‐ Effloreszenz: gelb‐orange Plaques ‐ Lokalisation: Kopfhaut, Gesicht inklusive Augenlider, Stamm, Axillae, obere Extremitäten	*Cladribin*: 6 Zyklen 0,14 mg/kg/Tag für 5 Tage pro Monat	Komplette Remission
	**(4) Progressiv systemisches XD, m, 67 Jahre** *Kutane Manifestation*: ‐ Effloreszenz: rötliche Papeln und Nodi ‐ Lokalisation: Kopfhaut, Gesicht inklusive Augenlider, Stamm, Intertrigines, Perianalregion *Systembeteiligung*: DI, Atemwege	*Cladribin*: 4 Zyklen 0,14 mg/kg/Tag für 5 Tage pro Monat	Partielle Remission kutane und systemische Manifestationen
	**(5) Persistentes XD, m, 46 Jahre** *Kutane Manifestation*: ‐ Effloreszenz: rot‐bräunliche Papeln und Nodi ‐ Lokalisation: Augenlider, Axillae	*Cladribin*: 5 Zyklen 0,14 mg/kg/Tag für 5 Tage pro Monat	Hohe partielle Remission (ca. 80 %)
Kim et al., 2010 *Fallbericht*	**Persistentes XD, w, 47 Jahre** *Kutane Manifestation*: ‐ Effloreszenz: gelb‐braune Knötchen und Papeln, teilweise zu Plaques konfluierend ‐ Lokalisation: Kopfhaut, Gesicht inklusive Augenlider, Mund‐ und Nasenschleimhaut, Hals, Schultern, Axillae, Perianalbereich	*Kombinationstherapie (sequenziell)*: ‐ operative Entfernung ‐ CO_2_‐Laser ‐ Prednisolon oral (20–40 mg/Tag für 11 Wochen, Ausschleichen über 10 Wochen)	Progression mukokutane Manifestationen
*Oka et al., 2010* *Fallbericht*	**Progressiv systemisches XD, m, 30 Jahre** *Kutane Manifestation*: ‐ Effloreszenz: multiple, gelb‐bräunliche Papeln, teilweise zu Plaques konfluierend ‐ Lokalisation: Gesicht inklusive Augenlider, Zunge, Hals, Intertrigines, Genitalien, Abdomen, Ellenbeugen, Kniekehlen ‐ Symptomatik: Juckreiz *Systembeteiligung*: DI, obere Atemwege, Konjunktiven	*Initial Prednisolon‐Monotherapie*:50 mg/Tag für 14 Tage, dann 40 mg/Tag für 14 Tage und 30 mg/Tag für 1 Monat*Kombinationstherapien (sequenziell)*: ‐ Clofibrat 1500 mg/Tag + Prednisolon 25 mg/Tag für 1 Monat ‐ Clofibrat + Dexamethason, initial 3 mg/Tag, über 5 Monate ‐ Clofibrat + Dexamethason + Etretinat 50 mg/Tag für 1 Monat ‐ Dexamethason 0,25 mg/Tag Monotherapie	Progression aller Manifestationen *Clofibrat + Dexamethason + Etretinat*: Remission an der Zunge, Stabilisierung der kutanen Manifestationen, Progression der ZNS‐Manifestationen
Yusuf et al., 2009 *Fallbericht*	**Progressiv systemisches XD, w, 32 Jahre** *Kutane Manifestation*: ‐ Effloreszenz: konfluierende Papeln, Plaques ‐ Lokalisation: Gesicht, Rumpf, Extremitäten *Systembeteiligung*: Kehlkopf und Pharynx, Polyurie, Polydipsie, Dysphonie	*Prednisolon*: ‐ 20 mg 2 x täglich für 22 Wochen	Teilremission mukokutaner Manifestationen, Kehlkopf und Pharynx Keine Veränderung Dysphonie
Eisendle et al., 2008 *Fallbericht*	**Progressiv systemisches XD, m 42 Jahre** *Kutane Manifestation*: ‐ Effloreszenz: gelb‐bräunliche Papeln ‐ Lokalisation: Gesicht inklusive Augenlider, Intertrigines, Ellenbeugen, perianal, Nasen‐Rachen‐Mundschleimhaut *Systembeteiligung*: Pharynx, Konjunktiven, ossäre Läsion (linker Unterarm)	*EtoposidOperative Versorgung*: Naseneingang, Handgelenk, Mundschleimhaut*Interferon‐γ*: s.c., 2 Mio. IU, 3 x/Woche für 3 Monate*Kombinationstherapie*: ‐ Rosiglitazon: 4 mg 1 x/Tag ‐ Simvastatin: 10 mg 1 x/Tag ‐ Acipimox: 250 mg 2 x/Tag	*Etoposid: k*eine Veränderung *Operation*: Remission der operierten Manifestationen *Interferon*: Remission kutaner Manifestationen, Rezidiv nach Absetzen *Kombinationstherapie*: Teilremission kutaner Manifestationen, Stabilisierung mukosaler und ossärer Manifestationen
Hisanaga et al., 2004 *Fallbericht*	**Progressiv systemisches XD, m, 68 Jahre** *Kutane Manifestation*: ‐ Effloreszenz: rötliche und gelb‐bräunliche bis zu 15 × 20 cm messende Plaques mit indurierten Rändern, vereinzelte Papeln ‐ Lokalisation: Rücken *Systembeteiligung*: Dickdarm, Rektum, Lunge, DI	*Kombinationstherapie*: ‐ systemische Steroide ‐ Antibiotika ‐ Antimykotika	Patient verstorben bei respiratorischer Insuffizienz infolge des XD

### Physikalische Therapieansätze

Die operative Entfernung kutaner Xanthome wurde in mehreren Arbeiten als alleinige oder ergänzende Maßnahme zur Reduktion der Symptomlast beschrieben.^6^, ^12^, ^13^ Das Auftreten neuer Läsionen konnte hierdurch oftmals nicht verhindert werden.^6^, ^13^ Operative Maßnahmen wurden auch im Rahmen notfallmäßiger Indikationen beschrieben, wie zur Verhinderung einer Verlegung der Atemwege bei Befall des Pharynx.^12^

In drei der eingeschlossenen Arbeiten wurden Laserbehandlungen im Gesicht vorgenommen.^6^, ^13^, ^14^ Der Einsatz einer 1450 nm‐Diodenlasertherapie in den betroffenen Gesichtspartien konnte kosmetisch überzeugende Ergebnisse erzielen, es wurden keine Rezidive oder Komplikationen beschrieben.^13^ In einem weiteren Fallbericht wurde ein CO_2_‐Laser zur Behandlung verwendet. Hierbei zeigte sich jedoch in der Folgeuntersuchung nach 21 Wochen ein Rezidiv.^6^ Eine kürzlich publizierte Arbeit berichtete über den erfolgreichen Einsatz des *Pulsed‐Dye*‐Lasers im Gesicht.^14^

Durch Schmalspektrum‐UVB‐Phototherapie konnte in einem Fall die pruriginöse Komponente einer persistenten XD gelindert werden, zudem wurde die Stabilisierung des Hautbefundes beschrieben.^15^

### Lipidsenkende Therapien

Der Einsatz lipidsenkender Therapien erfolgte in drei der eingeschlossenen Fallberichte.^8^, ^12^, ^16^ In allen diesen Fällen wurden Patienten mit ausgeprägter progressiver, systemischer Variante des XD behandelt.^8^, ^12^, ^16^ In zwei Fällen wurde zumindest zeitweise eine Kombination aus mehreren Lipidsenkern (Rosiglitazon, Simvastatin und Acipimox) angewandt.^8^, ^12^ Oka et al. kombinierten hingegen den Lipidsenker Clofibrat mit einer systemischen Steroidtherapie.^16^ Die Verwendung der Lipidsenker führte jeweils zur Stabilisierung oder Regredienz des Befunds.

### Immunsuppressive Therapien

Sowohl für die persistente kutane als auch für die systemische Variante des XD wurden in insgesamt zehn Arbeiten verschiedene immunsuppressive Therapien beschrieben.^6^, ^12^, ^16^, ^17^, ^18^, ^19^, ^20^, ^21^

Die Anwendung systemischer Glucocorticoide, wie Prednisolon oder Dexamethason, wurde für die persistente und progressive Form des XD vielfach berichtet.^6^, ^16^, ^17^, ^18^ Eine Remissionstendenz der Erkrankung wurde lediglich in Kombination mit anderen immunsuppressiven Medikamenten beschrieben.^17^ Auch die topische Anwendung allein scheint keine Besserung zu bewirken.^16^

Der Einsatz von Azathioprin in Kombination mit Prednisolon wurde in einem Fall berichtet und konnte im Beobachtungszeitraum von drei Monaten eine partielle Remission der Hautveränderungen erzielen.^17^ Der Calcineurininhibitor Ciclosporin hatte in einer Kasuistik keinen Einfluss auf den Hautbefund.^21^

Im Fall eines ausgeprägten progressiven, systemischen XD wurde der Interleukin‐1‐Rezeptorantagonist Anakinra eingesetzt, der zur Remission der Haut‐ und der ZNS‐Läsionen führte.^19^ Interferon‐γ wurde ebenfalls bei einem Patienten mit systemischem XD angewandt.^12^ Für die Dauer der Anwendung wurden eine Remission der Hautveränderungen und Stabilisierung der systemischen Beteiligung erzielt, nach Absetzen der Medikation kam es jedoch innerhalb von 3 Monaten zu erneutem Progress der Erkrankung.^12^

### Zytostatikabasierte Therapien

Zytostatikabasierte Therapien wurden in insgesamt neun Arbeiten berichtet.^2^, ^8^, ^12^, ^22^, ^23^, ^24^, ^25^, ^26^, ^27^ In den meisten Fällen waren die Patienten an progressivem, systemischem XD erkrankt.

Die Therapie mit Cladribin (Cd2‐A) wurde mit insgesamt zwölf Fällen in sechs verschiedenen Arbeiten am häufigsten berichtet.^2^, ^22^, ^23^, ^25^, ^26^, ^27^ Sieben Patienten waren an der progressiven systemischen Variante erkrankt und in fünf Fällen lag die persistente kutane Form vor. Das Patientenalter lag zwischen 20 und 67 Jahren. Alle Patienten erhielten an fünf aufeinanderfolgenden Tagen pro Monat eine Dosis von 0,14 mg/kg Körpergewicht (KG)/Tag über fünf bis neun Therapiezyklen. In allen berichteten Fällen ließ sich ein Progressionsstopp der kutanen Läsionen verzeichnen, mit zumindest partieller Remission. Vollständige Remissionen traten in drei der zwölf Fälle ein.^2^, ^4^ Remissionen wurden auch bei Patienten mit Schleimhautveränderungen sowie bei ZNS‐Beteiligung beschrieben.^2^, ^4^, ^27^ In allen Fällen konnte ein Progress der ZNS‐Beteiligung verhindert werden.^2^, ^22^, ^23^, ^27^

Unter Behandlung mit dem Tyrosinkinase‐Inhibitor Imatinib konnten die Hautveränderungen eines Patienten deutlich reduziert werden.^24^ Auch die DI‐assoziierten Beschwerden des Patienten nahmen ab, was als Hinweis auf eine zentrale Remission der Erkrankung bewertet wurde.^24^

Cyclophosphamid führte in einem Fallbericht über einen Zeitraum von 2 Jahren zur Stabilisierung des Befundes, jedoch ohne Anzeichen einer Remission.^8^ Etoposid erzielte ebenfalls einen Stillstand der Erkrankungsdynamik ohne Rückbildung der bestehenden Symptomatik.^12^

## Sonstige Therapieversuche

Der Einsatz von Doxycyclin über einen Monat konnte bei einem persistenten XD nicht zur Besserung des Hautbefundes beitragen.^21^ Ebenfalls erfolglos war die orale Gabe des Retinoids Etretinat bei einem Patienten mit progressiver systemischer XD.^16^

## DISKUSSION

Die vorliegende systematische Literaturrecherche ergab eine heterogene Datenlage zur Therapie des XD. Aufgrund der Seltenheit der Erkrankung sind die weltweit publizierten Arbeiten limitiert, es handelt sich hierbei um Fallberichte und Fallserien. Prospektive oder retrospektive Studien wurden bisher nicht publiziert. Diese Literaturübersicht kann daher für die Behandlung von Patienten mit XD eine wichtige und hilfreiche Grundlage darstellen.

In seiner persistenten Form stellt das XD eine rein dermatologische Erkrankung dar. Da Abheilungen spontan auftreten können, kann unter Umständen ein abwartendes Management geboten sein.^9^ Im Falle starker kosmetischer Beeinträchtigung kann neben der operativen Entfernung auch eine Laserbehandlung angeboten werden, wenngleich es sich hierbei um rein ablative beziehungsweise symptomatische Therapien handelt und somit das Risiko eines Rezidivs bedacht werden sollte.^13^

Die lipidsenkenden Medikamente Simvastatin, Rosiglitazon, Acipimox und Clofibrat erwiesen sich in mehreren Kasuistiken auch bei progressivem systemischem XD als wirksam.^8^, ^12^ Genutzt wird hier vor allem das indirekte antiinflammatorische Potenzial der Wirkstoffe. Der Wirkmechanismus beruht auf der Reduktion der Akkumulation entzündungsfördernder Lipide in den schaumzellartigen Histiozyten.^12^ Aufgrund des vergleichsweise günstigen Nebenwirkungsprofils bietet sich der Einsatz lipidsenkender Medikamente als Erstlinientherapie an, sofern keine Kontraindikationen bestehen. Ob die Kombination mehrerer lipidsenkender Wirkstoffe zur erfolgreichen Behandlung notwendig ist, kann anhand der aktuellen Datenlage nicht abschließend beurteilt werden.

Bei Patienten mit Schleimhautbefall sowie bei systemischer Ausprägung ist oftmals eine interdisziplinäre Versorgung notwendig. Der Befall des ZNS mit Entstehung eines DI gilt als mögliche Komplikation.^2^, ^23^ Bei DI können die Symptome in der Regel mit der ergänzenden Anwendung von Desmopressin suffizient kontrolliert werden.^27^ Da die entstehenden funktionellen Schäden als irreversibel gelten, sollte ein Progressionsstopp das primäre Ziel sein.^23^ Für das XD vom progressiven systemischen Typ wurde am häufigsten über den erfolgreichen Einsatz von Cd2‐A berichtet. ^2^, ^22^, ^23^, ^25^, ^26^, ^27^ Hierbei handelt es sich um ein synthetisches Purin‐Analogon, das die DNA‐Synthese und ‐Reparatur in T‐ und B‐Lymphozyten hemmt.^28^ Cd2‐A wird unter anderem zur Behandlung myeloproliferativer Erkrankungen eingesetzt, ein vermuteter Wirkmechanismus beim XD basiert auf der Ähnlichkeit von Monozyten und den beim XD vorliegenden Histiozyten.^2^ Unter Cd2‐A wurde neben der suffizienten Symptomkontrolle an der Haut zum Teil auch ein Rückgang von Schleimhautbeteiligungen beobachtet.^2^, ^22^, ^23^, ^27^

Auch der erfolgreiche Einsatz anderer Zytostatika und immunsupprimierender Medikamente wurde publiziert.^12^, ^19^, ^20^, ^24^, ^28^ Für den erfolgreichen Einsatz von Anakinra wurde die entzündungshemmende Wirkung auf Makrophagen und deren Proliferation zugrunde gelegt.^19^ Grundsätzlich sollte der Einsatz dieser Medikamente stets unter Abwägung des Nutzen‐Risiko‐Verhältnisses erfolgen, da potenziell schwerwiegende Nebenwirkungen auftreten können. Ausführliche Patientenaufklärung sowie sorgfältige Abwägung des Therapieeinsatzes in Abhängigkeit von Ausprägung und Symptomatik sollten obligat sein. Zudem ist zu beachten, dass es sich um erfolgreiche Berichte einzelner oder weniger Fälle handelt. Der Einsatz von Steroiden, Antibiotika und Retinoiden erscheint anhand der aktuellen Datenlage hingegen nicht empfehlenswert.^2^, ^22^, ^23^, ^27^

Eine mögliche Limitation dieser Arbeit im Hinblick auf die Vollständigkeit könnte sein, dass die Suche nur in einer Datenbank durchgeführt wurde. Jedoch handelt es sich bei hierbei um die gängigste medizinische Datenbank, weshalb davon ausgegangen werden kann, dass nahezu alle relevanten Arbeiten hier gefunden werden konnten. Nicht ausgeschlossen werden kann, dass relevante Studien durchgeführt, aber nicht veröffentlicht wurden (Publikationsbias).

Weitere Studien zum XD sind dringend erforderlich, um künftig evidenzbasierte Behandlungsrichtlinien entwickeln zu können.

## Schlussfolgerungen

Die vorliegende Literaturrecherche dient als Übersicht der bisher publizierten Therapieansätze für das Xanthoma disseminatum und kann in der Patientenbehandlung als Grundlage zur Therapieentscheidung herangezogen werden. Die chirurgische Exzision von Xanthomen ist mit einer hohen Rezidivrate verbunden, kann jedoch in bestimmten Fällen indiziert sein. Dies gilt vor allem für funktionell einschränkende Läsionen, beispielsweise bei Atemwegsverlegungen. Lipidsenker sollten als Erstlinientherapie bei Notwendigkeit einer Systemtherapie in Betracht gezogen werden. Hinsichtlich weiterer Systemtherapeutika besteht aktuell die beste Datenlage zu Cd2‐A. Bei ausgeprägten Befunden kann auch die Kombination verschiedener Maßnahmen erwogen werden.

Zusammenfassend können anhand der aktuellen Datenlage keine einschlägigen Empfehlungen formuliert werden. Dennoch sollte sich bei der Behandlung von Patienten mit XD an den bisher publizierten Fallberichten und Fallserien orientiert werden.

## DANKSAGUNG

Open access Veröffentlichung ermöglicht und organisiert durch Projekt DEAL.

## INTERESSENKONFLIKT

Keiner.
